# Plant Abiotic Stress Challenges from the Changing Environment

**DOI:** 10.3389/fpls.2016.01123

**Published:** 2016-07-27

**Authors:** Andy Pereira

**Affiliations:** Crop, Soil, and Environmental Sciences, University of ArkansasFayetteville, AR, USA

**Keywords:** abiotic stress, gene regulation, stress tolerance, epigenetics, transposons, genome wide association studies, stress signaling

The challenges of abiotic stress on plant growth and development are evident among the emerging ecological impacts of climate change (Bellard et al., 2012), and the constraints to crop production exacerbated with the increasing human population competing for environmental resources (Wallace et al., 2003). Climate change is predicted to affect agricultural production the most, primarily at low latitudes populated by developing countries, with adverse effects of increasing carbon dioxide and high temperature, challenging researchers toward devising adaptation strategies (Rosenzweig et al., 2014). These constraints to global food supply and a balanced environment encourage research and development of climate smart crops, resilient to climate change (Wheeler and Von Braun, 2013).

The field of plant abiotic stress encompasses all studies on abiotic factors or stressors from the environment that can impose stress on a variety of species (Sulmon et al., 2015). These stressors include extreme levels of light (high and low), radiation (UV-B and UV-A), temperature [high and low (chilling, freezing)], water (drought, flooding, and submergence), chemical factors (heavy metals and pH), salinity due to excessive Na^+^, deficient or in excess of essential nutrients, gaseous pollutants (ozone, sulfur dioxide), mechanical factors, and other less frequently occurring stressors. Since combinations of these stresses such as heat and drought frequently occur under field conditions, and can cause unique effects that cannot be predicted from individual stressors (Suzuki et al., 2014), a multiplicity of physiological interactions can be expected, needing individual novel solutions.

Plants are rooted in the environment they grow in, and have to adapt to the changing conditions brought about by the multitude of environmental factors, with extreme levels eliciting abiotic stress. A grand challenge in abiotic stress biology is to decipher how plants perceive the different stressors, how the early signals are transduced within the plant, what is the diversity of response pathways elicited by them, and how are they genetically determined (Yoshida et al., 2014). Beyond model plants and reference genotypes, the challenge is to identify how signaling pathways have evolved within a species to program a suite of responses differing in signals and regulatory networks, and constitute genotypes that are adapted to specific stressful environments. Many studies have begun to deal with the comparison of a few genotypes, such as tolerant and sensitive within a species, for the analysis of differential responses to a defined stress. Since these responses can be due to differences in sets of genes, an understanding of the diversity in signaling pathways can come only by making a systems level study of the differences between genotypes. Such comparative studies offer a challenge for the integration of diverse functional genomics datasets of gene expression, metabolomics, and stress physiological responses to make comparisons in the network of responses across genotypes.

A compatible environment for one plant genotype may not be for another, and all external factors abiotic or biotic, can raise a challenge or stress to the plant depending on the plants genetic constitution and adaptive response. The specific genotype × environment interaction combinations offer multitude of effects in response to the environment (Des Marais et al., 2013). Molecular genetic analysis of specific genes conferring stress tolerance from tolerant crop accessions have resulted in the map-based isolation of genes for submergence tolerance (Xu et al., 2006) and salt tolerance (Ren et al., 2005) in rice, among many others. The challenge ahead is in the analysis of natural variation in populations using genome wide association studies (GWAS) to dissect quantitative traits from field screens of diverse genotypes and map specific naturally occurring “stress tolerant loci.” This has been successful for salt tolerance (Kumar et al., 2015) in rice, and also led to the identification from maize of the first drought tolerance gene (Mao et al., 2015). The variation within the maize drought tolerance gene is particularly interesting because the drought sensitive allele contains a transposon insert in the promoter that is involved in epigenetic regulation of the gene that differs in distribution between temperate and tropical maize. Transposons as agents of regulation of genes in abiotic stress are being identified in maize as “controlling elements” involved in the regulation of around 20% of the abiotic stress responsive genes (Makarevitch et al., 2015), indicating evolution and selection of novel stress protective alleles active in natural populations. Similarly in rice, insertions of the mPing transposon with insertion preference in the 5′ regions of genes were shown to up-regulate the downstream genes and render them stress responsive (Naito et al., 2009). The intriguing challenge ahead is to now see how far McClintock's controlling elements (McClintock, 1984), that are induced to move under stress can help plants survive abiotic stresses by creating and regulating networks of genes for stress protection.

The new challenges will come from genome-wide analyses of stress tolerant genotypes from multiple plant species that will probably reveal novel tolerance and selective mechanisms in natural populations. The supporting technologies from next-generation sequencing to GWAS are available in many plant species, and much research is concentrated in this area for stress tolerance. This is therefore an area for future discoveries that will reveal the evolution of diverse mechanisms for stress tolerance that could be valuable for the design of crop improvement strategies including for climate change challenges.

A fruitful strategy for the identification of stress tolerance genes has been by reverse genetics analyses of candidate genes identified through gene expression studies and other bioinformatics methods. The biological role of such candidate genes has been most often tested by the analysis of overexpression, knockout/knockdown genotypes in model, and crop plants (Todaka et al., 2015). Overexpression studies with transcription factors and other regulatory genes have been popular in transgenic crops, with the objective of improving their stress tolerance and productivity (Mickelbart et al., 2015), and often enabling applications across plant species. The potential redundancy of stress tolerance genes remains a challenge, since overexpression studies might not represent the natural function of genes in the plant. Nevertheless, all studies testing the potential phenotype of genes for alterations in stress response provide useful information on the gene function as well as the applications. Gene expression analysis of plants in response to abiotic stresses reveals a large fraction of the genome can be perturbed, reflecting the plasticity in stress response and protection. The complexity of a plants' response to abiotic stress factors, in interaction with its genetic constitution, provides a multitude of morpho-physiological, biochemical, gene expression, and other molecular responses that can best be described by networks of response pathways leading to expression of tolerance and adaptation to the environment.

The role of genetics and evolution propounded by Darwinism seemed to prevail over the opposing views of Lamarckism, proposing life forms could acquire information from their environment and pass it on in their genes. Now, two centuries later the evidence from epigenetics is showing us in surprising detail, with the sophistication of genomics technologies, how the epigenome carries information that is not encoded in the DNA to offspring, and can even provide a mechanism for acclimation and adaptation to stress (Avramova, 2015). The role of the environment, and subsequently stresses that might permanently plague plants, probably have a significant epigenetic influence on the behavior of plants and their progeny, and provide new challenges to re-visit plant–environment interactions.

Abiotic stresses will remain a challenge to the natural environment and agriculture. The early evolution of land plants took place under dry conditions with extremes of temperature and harsh sunlight, while crop domestication occurred later in more favorable environments. Subsequently, the selection of plants for productivity traits did not always result in crops that are productive under random stress factors, although the natural variation of crops are genetic reservoirs for abiotic stress adaptation. Presently, with the competing uses of land and the growing world population we are challenged to produce more in less area with dwindling resources of water, confronted with climate change increases in temperature and carbon dioxide, and the unpredictable local microclimate adversely affecting crop productivity. The challenges before us in plant biology and crop improvement are to integrate the systems level information on abiotic stress response pathways, identify stress protective networks, and engineer environmentally stable crops that yield more, with less water and dwindling natural resources, to feed the growing world population.

## Author contributions

The author confirms being the sole contributor of this work and approved it for publication.

## Funding

This work was supported in part by the National Science Foundation awards DBI-0922747 and ABI1062472, and by the National Research Initiative Competitive Grant No. VAR-2008-01133 from the USDA National Institute of Food and Agriculture.

### Conflict of interest statement

The author declares that the research was conducted in the absence of any commercial or financial relationships that could be construed as a potential conflict of interest.
